# Supplementary material for: Gene signatures with predictive and prognostic survival values in human osteosarcoma
Source: PeerJ. 2021 Jan 15;9:e10633. doi: 10.7717/peerj.10633 (PMC7812922; doi:10.7717/peerj.10633)
Supplement: Supplemental Information 1 [file peerj-09-10633-s001.zip › Supplementary tables/5.table_result/Table 1_lasso model.docx]

Table 1 Lasso model

| Ensemble ID | Gene | Chromosome location | Coefficient of lasso model | HR | pvalue |
| --- | --- | --- | --- | --- | --- |
| ENSG00000103274.9 | NUBP1 | chr16:10743786-10769351:(+) | -0.2216 | 0.905 (0.86, 0.953) | 0.000 |
| ENSG00000163219.10 | ARHGAP25 | chr2:68679601-68826833:(+) | -0.0503 | 0.795 (0.685, 0.924) | 0.003 |
| ENSG00000077420.14 | APBB1IP | chr10:26438203-26567803:(+) | -0.0429 | 0.924 (0.88, 0.97) | 0.001 |
| ENSG00000162517.11 | PEF1 | chr1:31629862-31644896:(-) | -0.0230 | 0.979 (0.966, 0.992) | 0.002 |
| ENSG00000102226.8 | USP11 | chrX:47232690-47248328:(+) | -0.0123 | 0.982 (0.97, 0.994) | 0.004 |
| ENSG00000179163.11 | FUCA1 | chr1:23845077-23868294:(-) | -0.0104 | 0.969 (0.949, 0.99) | 0.003 |
| ENSG00000189171.12 | S100A13 | chr1:153618787-153634092:(-) | 0.0019 | 1.007 (1.004, 1.011) | 0.000 |
| ENSG00000132535.17 | DLG4 | chr17:7189890-7219702:(-) | 0.0088 | 1.064 (1.021, 1.109) | 0.003 |
| ENSG00000158315.9 | RHBDL2 | chr1:38885807-38941799:(-) | 0.0144 | 1.01 (1.006, 1.013) | 0.000 |
| ENSG00000176171.10 | BNIP3 | chr10:131966455-131981931:(-) | 0.0170 | 1.009 (1.004, 1.014) | 0.000 |
| ENSG00000167549.17 | CORO6 | chr17:29614756-29622907:(-) | 0.0425 | 1.054 (1.021, 1.088) | 0.001 |
| ENSG00000125337.15 | KIF25 | chr6:167996241-168045089:(+) | 0.0570 | 1.148 (1.076, 1.224) | 0.000 |
| ENSG00000179262.8 | RAD23A | chr19:12945855-12953642:(+) | 0.0741 | 1.005 (1.002, 1.008) | 0.000 |
| ENSG00000147378.10 | FATE1 | chrX:151716035-151723194:(+) | 0.0862 | 2.217 (1.518, 3.238) | 0.000 |
| ENSG00000113739.9 | STC2 | chr5:173314713-173329503:(-) | 0.0895 | 1.016 (1.005, 1.027) | 0.004 |
| ENSG00000197467.12 | COL13A1 | chr10:69801931-69964275:(+) | 0.0910 | 1.021 (1.013, 1.029) | 0.000 |
| ENSG00000017483.13 | SLC38A5 | chrX:48458537-48470256:(-) | 0.1003 | 1.008 (1.003, 1.014) | 0.002 |
| ENSG00000136997.13 | MYC | chr8:127735434-127741434:(+) | 0.1635 | 1.003 (1.002, 1.005) | 0.000 |
| ENSG00000138028.13 | CGREF1 | chr2:27098889-27119115:(-) | 0.2105 | 1.015 (1.009, 1.021) | 0.000 |
| ENSG00000241563.3 | CORT | chr1:10449719-10451902:(+) | 0.2504 | 1.026 (1.016, 1.036) | 0.000 |

HR: Hazard Ratio; CI: Confidence Interval
