# Supplementary material for: Gene signatures with predictive and prognostic survival values in human osteosarcoma
Source: PeerJ. 2021 Jan 15;9:e10633. doi: 10.7717/peerj.10633 (PMC7812922; doi:10.7717/peerj.10633)
Supplement: Supplemental Information 1 [file peerj-09-10633-s001.zip › Supplementary tables/5.table_result/Table 2.docx]

| Characters | Level | Low risk | High risk | P value |
| --- | --- | --- | --- | --- |
| n |  | 42 | 42 |  |
| Gender | Female (%) | 16 ( 38.1) | 21 (50.0) | 0.379 |
|  | Male (%) | 26 ( 61.9) | 21 (50.0) |  |
| Age | <18 (%) | 32 ( 76.2) | 34 (81.0) | 0.791 |
|  | >=18 (%) | 10 ( 23.8) | 8 (19.0) |  |
| Overall survival | Alived (%) | 42 (100.0) | 13 (31.0) | <0.001 |
|  | Dead (%) | 0 ( 0.0) | 29 (69.0) |  |
| Recurrence | No (%) | 32 ( 76.2) | 13 (31.0) | <0.001 |
|  | Yes (%) | 10 ( 23.8) | 29 (69.0) |  |
| Metastasis | No (%) | 37 ( 88.1) | 26 (61.9) | 0.011 |
|  | Yes (%) | 5 ( 11.9) | 16 (38.1) |  |
| Primary tumor site | Arm/Hand OR Pelvis (%) | 3 ( 7.1) | 5 (11.9) | 0.713 |
|  | Leg/Foot (%) | 39 ( 92.9) | 37 (88.1) |  |
| Specific tumor site | Femur (%) | 13 ( 31.0) | 25 (59.5) | 0.015 |
|  | Tibia or others (%) | 29 ( 69.0) | 17 (40.5) |  |

Table 2
