# Supplementary material for: Gene signatures with predictive and prognostic survival values in human osteosarcoma
Source: PeerJ. 2021 Jan 15;9:e10633. doi: 10.7717/peerj.10633 (PMC7812922; doi:10.7717/peerj.10633)
Supplement: Supplemental Information 3 [file peerj-09-10633-s003.docx]

| Module colors | Gene frequency |
| --- | --- |
| black | 171 |
| blue | 501 |
| brown | 325 |
| green | 239 |
| grey | 1009 |
| magenta | 129 |
| pink | 149 |
| purple | 111 |
| red | 195 |
| turquoise | 1922 |
| yellow | 249 |

Table 4 Eleven co-expression modules with WGCNA analysis
