# Supplementary material for: Gene signatures with predictive and prognostic survival values in human osteosarcoma
Source: PeerJ. 2021 Jan 15;9:e10633. doi: 10.7717/peerj.10633 (PMC7812922; doi:10.7717/peerj.10633)
Supplement: Supplemental Information 4 [file peerj-09-10633-s004.docx]

Table 5 magenta-module GO kegg, Gene ontology analysis of that are involved with the genes in the magenta co-expression module

| Category | ID | Description | GeneRatio | pvalue | p.adjust | qvalue | geneID | Count |
| --- | --- | --- | --- | --- | --- | --- | --- | --- |
| BP | GO:0002495 | antigen processing and presentation of peptide antigen via MHC class II | 14/120 | 3.0281E-15 | 3.60666E-12 | 2.76907E-12 | CD74/HLA-DRA/HLA-DRB1/HLA-DPB1/CTSL/HLA-DRB5/LGMN/HLA-DPA1/HLA-DMA/CTSS/TREM2/HLA-DQB1/AP1B1/HLA-DQA1 | 14 |
| BP | GO:0002504 | antigen processing and presentation of peptide or polysaccharide antigen via MHC class II | 14/120 | 3.49119E-15 | 3.60666E-12 | 2.76907E-12 | CD74/HLA-DRA/HLA-DRB1/HLA-DPB1/CTSL/HLA-DRB5/LGMN/HLA-DPA1/HLA-DMA/CTSS/TREM2/HLA-DQB1/AP1B1/HLA-DQA1 | 14 |
| BP | GO:0048002 | antigen processing and presentation of peptide antigen | 17/120 | 4.55386E-15 | 3.60666E-12 | 2.76907E-12 | CD74/HLA-DRA/HLA-DRB1/HLA-DPB1/CTSL/HLA-DRB5/LGMN/HLA-DPA1/HLA-DMA/CTSS/CYBB/TREM2/HLA-DQB1/AP1B1/HLA-DQA1/PSMB10/ERAP2 | 17 |
| BP | GO:0019886 | antigen processing and presentation of exogenous peptide antigen via MHC class II | 13/120 | 5.58015E-14 | 3.31461E-11 | 2.54484E-11 | CD74/HLA-DRA/HLA-DRB1/HLA-DPB1/CTSL/HLA-DRB5/LGMN/HLA-DPA1/HLA-DMA/CTSS/HLA-DQB1/AP1B1/HLA-DQA1 | 13 |
| BP | GO:0019882 | antigen processing and presentation | 17/120 | 8.8692E-14 | 4.21464E-11 | 3.23586E-11 | CD74/HLA-DRA/HLA-DRB1/HLA-DPB1/CTSL/HLA-DRB5/LGMN/HLA-DPA1/HLA-DMA/CTSS/CYBB/TREM2/HLA-DQB1/AP1B1/HLA-DQA1/PSMB10/ERAP2 | 17 |
| CC | GO:0042613 | MHC class II protein complex | 9/122 | 1.08624E-16 | 2.368E-14 | 1.5436E-14 | CD74/HLA-DRA/HLA-DRB1/HLA-DPB1/HLA-DRB5/HLA-DPA1/HLA-DMA/HLA-DQB1/HLA-DQA1 | 9 |
| CC | GO:0042611 | MHC protein complex | 9/122 | 1.85158E-14 | 2.01822E-12 | 1.31559E-12 | CD74/HLA-DRA/HLA-DRB1/HLA-DPB1/HLA-DRB5/HLA-DPA1/HLA-DMA/HLA-DQB1/HLA-DQA1 | 9 |
| CC | GO:0030669 | clathrin-coated endocytic vesicle membrane | 9/122 | 5.82369E-12 | 2.56644E-10 | 1.67296E-10 | CD74/HLA-DRA/APOE/HLA-DRB1/HLA-DPB1/HLA-DRB5/HLA-DPA1/HLA-DQB1/HLA-DQA1 | 9 |
| CC | GO:0071556 | integral component of lumenal side of endoplasmic reticulum membrane | 8/122 | 6.5563E-12 | 2.56644E-10 | 1.67296E-10 | CD74/HLA-DRA/HLA-DRB1/HLA-DPB1/HLA-DRB5/HLA-DPA1/HLA-DQB1/HLA-DQA1 | 8 |
| CC | GO:0098553 | lumenal side of endoplasmic reticulum membrane | 8/122 | 6.5563E-12 | 2.56644E-10 | 1.67296E-10 | CD74/HLA-DRA/HLA-DRB1/HLA-DPB1/HLA-DRB5/HLA-DPA1/HLA-DQB1/HLA-DQA1 | 8 |
| MF | GO:0042277 | peptide binding | 15/115 | 8.02809E-10 | 1.29008E-07 | 1.04733E-07 | CD74/HLA-DRA/APOE/HLA-DRB1/C1QA/HLA-DPB1/CD14/HLA-DRB5/HLA-DPA1/RAMP2/ITGB2/TREM2/HLA-DQB1/HLA-DQA1/ERAP2 | 15 |
| MF | GO:0042605 | peptide antigen binding | 7/115 | 9.40456E-10 | 1.29008E-07 | 1.04733E-07 | HLA-DRA/HLA-DRB1/HLA-DPB1/HLA-DRB5/HLA-DPA1/HLA-DQB1/HLA-DQA1 | 7 |
| MF | GO:0033218 | amide binding | 16/115 | 1.26478E-09 | 1.29008E-07 | 1.04733E-07 | CD74/HLA-DRA/APOE/HLA-DRB1/C1QA/PLTP/HLA-DPB1/CD14/HLA-DRB5/HLA-DPA1/RAMP2/ITGB2/TREM2/HLA-DQB1/HLA-DQA1/ERAP2 | 16 |
| MF | GO:0032395 | MHC class II receptor activity | 4/115 | 3.44795E-07 | 2.63768E-05 | 2.14136E-05 | HLA-DRA/HLA-DPA1/HLA-DQB1/HLA-DQA1 | 4 |
| MF | GO:0023026 | MHC class II protein complex binding | 4/115 | 2.89948E-06 | 0.000177448 | 0.000144059 | CD74/HLA-DRA/HLA-DRB1/HLA-DMA | 4 |
| KEGG | hsa05150 | Staphylococcus aureus infection | 16/78 | 4.66891E-16 | 6.58317E-14 | 4.96379E-14 | HLA-DRA/HLA-DRB1/C1QC/C1QA/C1QB/HLA-DPB1/HLA-DRB5/HLA-DPA1/HLA-DMA/ITGB2/FCGR3A/HLA-DQB1/FCGR2A/FPR3/HLA-DQA1/C3AR1 | 16 |
| KEGG | hsa05140 | Leishmaniasis | 13/78 | 2.79988E-13 | 1.56716E-11 | 1.18166E-11 | HLA-DRA/HLA-DRB1/HLA-DPB1/HLA-DRB5/HLA-DPA1/HLA-DMA/IFNGR1/ITGB2/CYBB/FCGR3A/HLA-DQB1/FCGR2A/HLA-DQA1 | 13 |
| KEGG | hsa04612 | Antigen processing and presentation | 13/78 | 3.33438E-13 | 1.56716E-11 | 1.18166E-11 | CD74/HLA-DRA/HLA-DRB1/CTSB/HLA-DPB1/CTSL/HLA-DRB5/LGMN/HLA-DPA1/HLA-DMA/CTSS/HLA-DQB1/HLA-DQA1 | 13 |
| KEGG | hsa04514 | Cell adhesion molecules (CAMs) | 15/78 | 7.11238E-12 | 2.50711E-10 | 1.8904E-10 | HLA-DRA/HLA-DRB1/HLA-DPB1/HLA-DRB5/HLA-DPA1/HLA-DMA/ESAM/ITGB2/PECAM1/HLA-DQB1/CDH5/CLDN5/HLA-DQA1/VCAM1/CD34 | 15 |
| KEGG | hsa04145 | Phagosome | 15/78 | 1.16021E-11 | 3.27179E-10 | 2.46697E-10 | HLA-DRA/HLA-DRB1/HLA-DPB1/CD14/CTSL/HLA-DRB5/HLA-DPA1/HLA-DMA/ITGB2/CTSS/CYBB/FCGR3A/HLA-DQB1/FCGR2A/HLA-DQA1 | 15 |
